# Supplementary figures and images for: Coxiella burnetii infects osteoclasts and alters their differentiation and function in a type IV secretion system-dependent manner
Source: Front Immunol. 2026 Jan 23;16:1724684. doi: 10.3389/fimmu.2025.1724684 (PMC12876216; doi:10.3389/fimmu.2025.1724684)

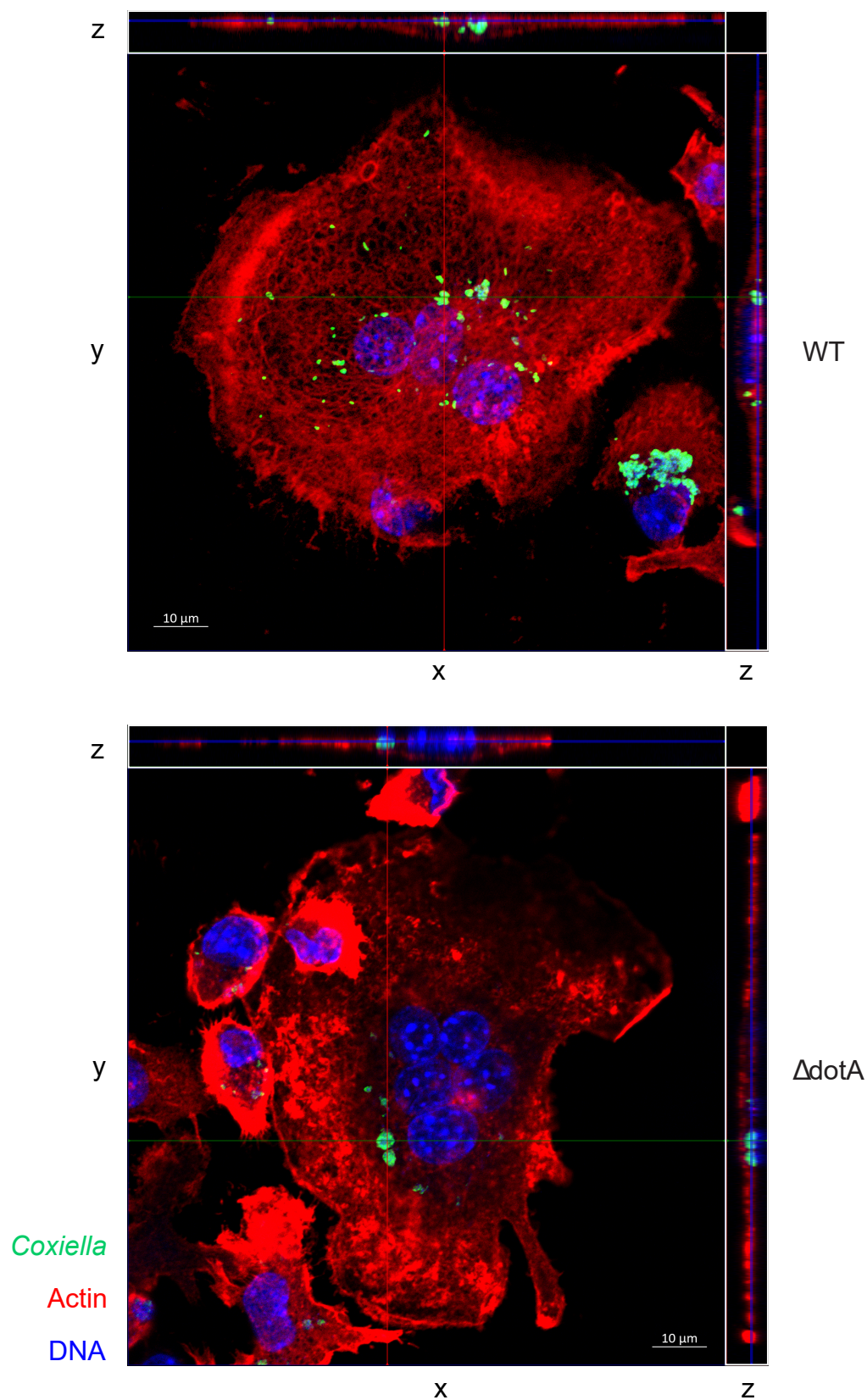

**Supplementary Figure 1**

Supplement: Supplementary Figure 1 — Phagocytosis of C. burnetii by myeloid cells and osteoclasts. Osteoclasts were differentiated from bone marrow progenitor cells in presence of M-CSF and RANKL. Cells were then infected after 2 days of differentiation with C. burnetii WT or ΔdotA at MOI 10 for 6 h. Gentamicin (200 µg/mL) was added for 1h to close the pulse infection. After 24 hpi, cells were stained for Coxiella (green - Alexa488), actin (red – Alexa647) and DNA (blue - DAPI) and imaged by fluorescent CLSFM (n=3). Orthogonal projections are depicted on top (x, z) and on the right side (y, z) of the top view (x, y) The scale bar represents 10 µm. [file Image1.pdf]

**A**

n.i.

WT

 $\Delta dotA$ 

72 hpi

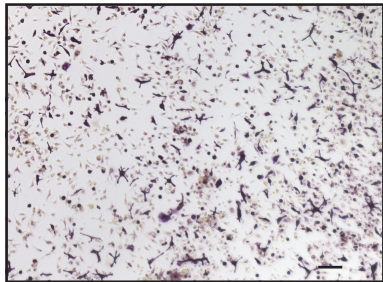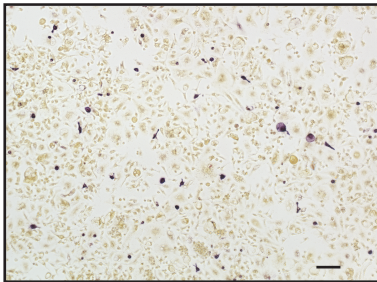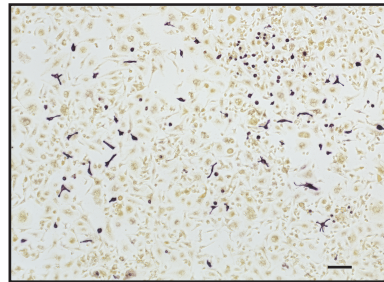

120 hpi

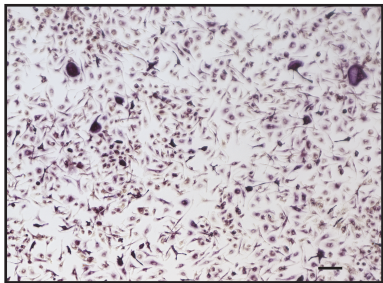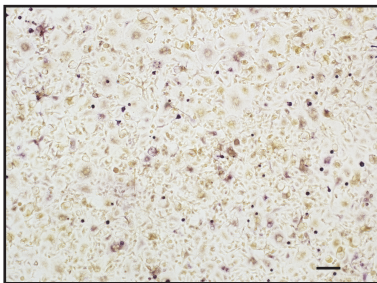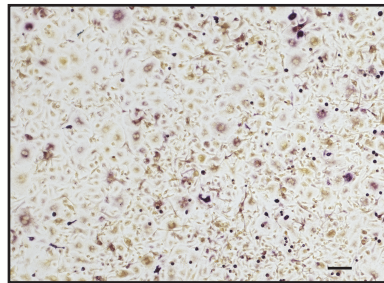**Supplementary Figure 3**

Supplement: Supplementary Figure 3 — C. burnetii infection of myeloid progenitor cells inhibits osteoclast differentiation (part 2). Osteoclast progenitors were infected with C. burnetii WT or ΔdotA before being stimulated with RANKL (6 hpi). Osteoclast differentiation was analyzed 72 hpi and 120 hpi (matching time point with the previous experiment presented in Figure 3A) by staining for TRAP. Cells were analyzed by light microscopy. Scale bar represents 100 μm. [file Image3.pdf]

**A**

n.i.

WT

 $\Delta dotA$ 

72 hpi

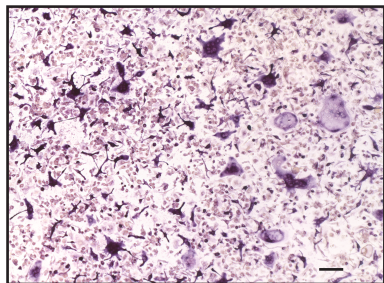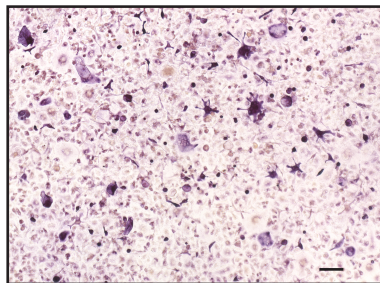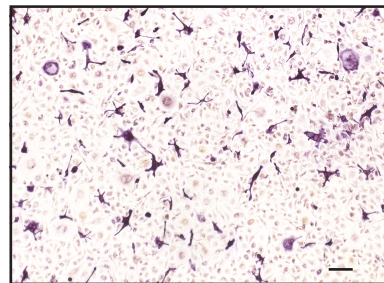 $\Delta ankG$  $\Delta caeB$ 

HK

72 hpi

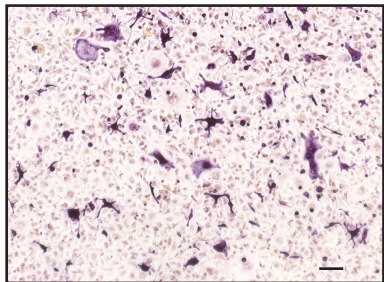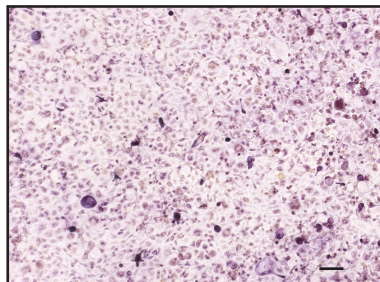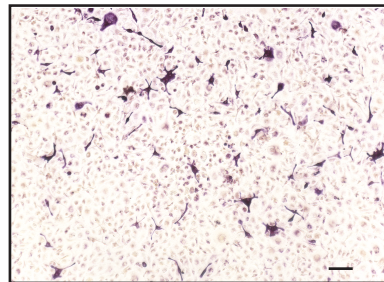**Supplementary Figure 4**

Supplement: Supplementary Figure 4 — Effector proteins of C. burnetii affect osteoclast differentiation (part 2). Osteoclast cultures were infected with C. burnetii WT, ΔdotA, ΔankG, ΔcaeB or heat-killed WT (HK) after 2 days of RANKL stimulation. Uninfected osteoclasts (n.i.) were used as control. Osteoclast differentiation was quantified 72 hpi by staining for TRAP. Cells were analyzed by light microscopy. Scale bar represents 100 μm. [file Image4.pdf]
