# Supplementary material for: Coxiella burnetii infects osteoclasts and alters their differentiation and function in a type IV secretion system-dependent manner
Source: Front Immunol. 2026 Jan 23;16:1724684. doi: 10.3389/fimmu.2025.1724684 (PMC12876216; doi:10.3389/fimmu.2025.1724684)
Supplement: Supplementary Figure 2 — Analysis of the correlation between the different approaches to quantify bacterial burden. (A) Osteoclasts were differentiated from bone marrow progenitor cells in presence of M-CSF and RANKL. Cells were then infected after 2 days of differentiation with C. burnetii WT or ΔdotA at MOI ranging from 1.25 to 20 for 6 h. Gentamicin (200 µg/mL) was added for 1h to close the pulse infection. After 24 hpi, genomic equivalent (GE) of C. burnetii per host cell were quantified by qPCR. Simple linear regression between GE per cell and MOI is depicted by a black line. 95% confidence interval is depicted with dashed lines. (B) In parallel, the bacterial burden was quantified by immunofluorescence staining on osteoclast cultures similarly treated. Briefly, cells were stained for Coxiella (green - Alexa488), actin (red – Alexa647) and DNA (blue - DAPI) and imaged by fluorescent CLSFM (n=3). The integrated Coxiella density staining per cell area was quantified using ImageJ. Simple linear regression between integrated Coxiella density staining per cell area and GE of C. burnetii per host cells is depicted by a black line. 95% confidence interval is depicted with dashed lines. [file Image2.pdf]

A

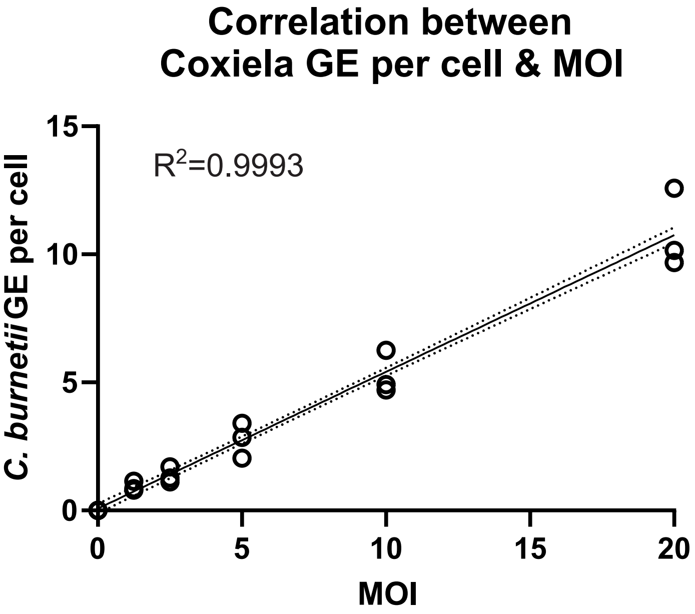

B

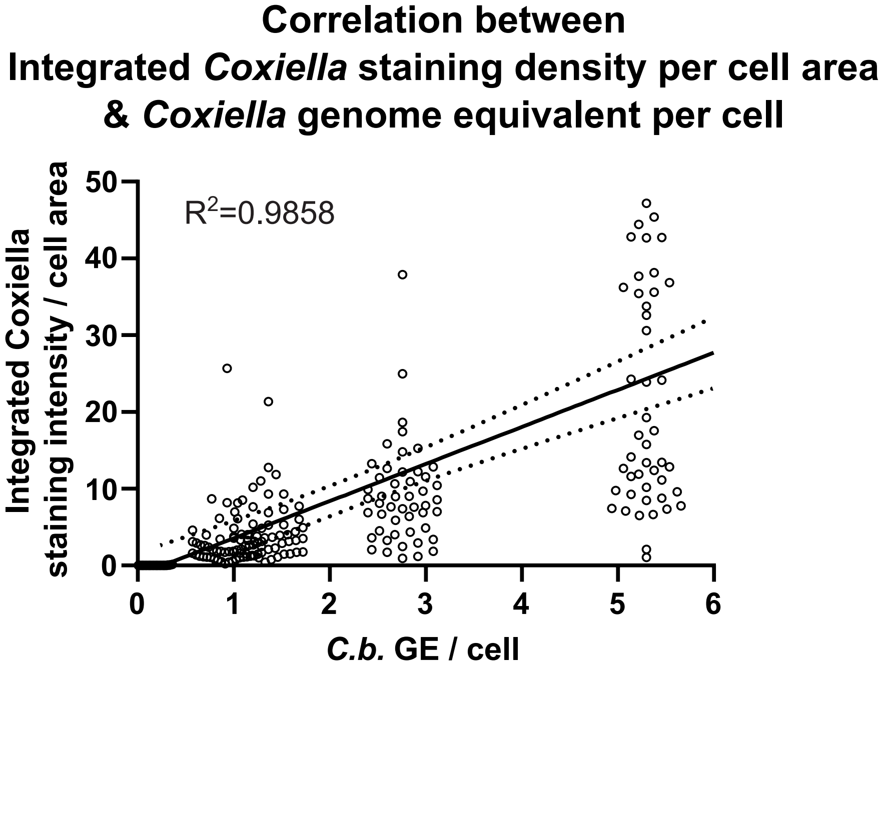

Supplementary Figure 2
